# Functional brain network mechanism of executive control dysfunction in temporal lobe epilepsy

**DOI:** 10.1186/s12883-020-01711-6

**Published:** 2020-04-15

**Authors:** Yanping Ren, Liping Pan, Xueyun Du, Yuying Hou, Xun Li, Yijun Song

**Affiliations:** Department of Neurology, Tianjin Medical University General Hospital, Key Laboratory of Neurotrauma, Variation and Regeneration, Ministry of Education and 4Tianjin Municipal Government, Tianjin Neurological Institute, Tianjin, 300052 China

**Keywords:** Temporal lobe epilepsy, Functional connectivity, Executive control, Theta, Scalp electroencephalogram

## Abstract

**Background:**

Executive control dysfunction is observed in a sizable number of patients with temporal lobe epilepsy (TLE). Neural oscillations in the theta band are increasingly recognized as having a crucial role in executive control network. The purpose of this study was to investigate the alterations in the theta band in executive control network and explore the functional brain network mechanisms of executive control dysfunction in TLE patients.

**Methods:**

A total of 20 TLE patients and 20 matched healthy controls (HCs) were recruited in the present study. All participants were trained to perform the executive control task by attention network test while the scalp electroencephalogram (EEG) data were recorded. The resting state signals were collected from the EEG in the subjects with quiet and closed eyes conditions. Functional connectivity among EEGs in the executive control network and resting state network were respectively calculated.

**Results:**

We found the significant executive control impairment in the TLE group. Compared to the HCs, the TLE group showed significantly weaker functional connectivity among EEGs in the executive control network. Moreover, in the TLE group, we found that the functional connectivity was significantly positively correlated with accuracy and negatively correlated with EC_effect. In addition, the functional connectivity of the executive control network was significantly higher than that of the resting state network in the HCs. In the TLE group, however, there was no significant change in functional connectivity strengths between the executive control network and resting state network.

**Conclusion:**

Our results indicate that the decreased functional connectivity in theta band may provide a potential mechanism for executive control deficits in TLE patients.

## Background

Cognitive impairment is one of the common comorbidities in patients with epilepsy, especially the temporal lobe epilepsy (TLE) [1, 2]. Execution control (EC) is the ability to monitor and resolve conflicts in the presence of competitive information. It is a sub-network of attention networks, and studies have found that EC function is often impaired in TLE patients [3–5]. In the EC network, the frontal-parietal network regulates the brain’s immediate information processing, and the cingulate-cap network provides a stable “establishment and maintenance state” throughout the process [6]. Temporal lobe epilepsy combined with EC deficits, greatly affects patients’ drug compliance, seizure frequency and prognosis, further reducing patients’ quality of life and increasing suicide risk [7]. Given the impact of EC deficits on the overall quality of life, it is important to understand the neural mechanisms of EC dysfunction in TLE.

In recent years, more and more evidence supports the network model of TLE, and in 2010 the International League Against Epilepsy first proposed that epilepsy is a type of brain network disease [8]. With the development of neuroimaging and neuroelectrophysiology, it is possible for connectomics to study nervous system diseases at the network level [9]. The in-depth study of brain network can have a better understanding of network diseases such as TLE. As a high-level cognitive function, EC is considered to be one of the cognitive functions closely related to daily life, including initiation, planning, organization and decision-making [10]. The EC network is responsible for EC [5]. In the EC network, the medial frontal cortex, anterior cingulate cortex, dorsolateral prefrontal cortex and parietal cortical are activated [5, 11–13]. Therefore, from the perspective of a brain network, studying TLE combined with EC dysfunction can better explain its neural mechanism.

The mechanism of networked brain research includes two levels: brain structure network and neural signal network [14]. The neural signal network studies the interconnection and functional integration of neural signals when the brain is in a certain functional state, including: functional connectivity and effective connectivity [15, 16]. Functional connectivity (FC) demonstrates significant temporal variability and dynamic reconfiguration during the performance of the neuropsychology tasks. Granger causal analysis [17] is an effective method to study the neural network of brain signals [18, 19]. Through the causal analysis of multi-channel neural signals, the connection mode of neural signal networks is calculated, and the neural signal network is constructed to study the cognitive brain network mechanism. The directional transfer function (DTF), characterized by high flexibility and easiness of implementation, is regarded as an effective way to evaluate the human brain FC among neural signals [20–22]. Zhang et al. constructed a functional brain network of working memory by calculating the DTF of EEG signals to study the working memory load of normal adults [22]. Wilke et al. calculated the source of information and its conduction direction during seizures by calculating DTF [20]. The EEG data structure is diverse, the frequency band is complex, the direction of computing function connection needs to be considered, and DTF has the advantage of processing complex structure EEG data [23].

The electrical activity of neurons and neuron clusters is the basis of cortical excitability, which is closely related to sensory, perceptual and cognitive activities. It is well established that the frontal theta activity is increased during high levels of cognitive demand [24, 25]. It is found that the power of theta band increased after EC behavioral task response, which indicated that theta oscillation has a role in long-range communications of cognitive processing [26]. Moreover, theta oscillation can promote post cognitive processing and environmental renewal between brain regions [27]. Our previous research shows that the power of the theta band in the EC of the healthy controls group is significantly higher than that of other bands, which is the dominant frequency band for performing EC function [28]. Compared with the HCs, the power of the theta band in the EC of the TLE group was significantly reduced. Nonetheless, what is the mechanism of theta oscillation absence from the view of brain network?

According to the above question, in the present study, we simultaneously recorded the EC behavioral data and EEGs signals to investigate the defect of functional brain network in TLE patients. Then, we calculated the FC strengths of the theta band in the HCs and TLE groups and the conduction direction of the FC in the dominant brain regions. The study aims to investigate the mechanism of brain network in theta oscillation absence among EEGs in EC dysfunction in TLE patients.

## Methods

### Participants

This study was approved by the ethics committee of Tianjin Medical University General Hospital, and informed consent was obtained from all participants. Twenty TLE patients and 20 HCs met the inclusion/exclusion criteria for the study. A subset of the subjects was included in a previous study by Li et al. [28]. All TLE patients were recruited between January 2016 and January 2017 via the Tianjin Medical University General Hospital, Tianjin, China. All the patients underwent a comprehensive assessment including neuropsychological and clinical symptoms and findings from Magnetic Resonance Imaging (MRI) and EEG examinations. The patients showed either no abnormalities or only hippocampal sclerosis on MRI; EEG results showed that the epileptogenic focus was located in the temporal lobe. Exclusion criteria included the follow: low compatibility or inability to complete the whole experimental process; alcohol dependence or drug abuse; suffering from other neurological or mental disorders, or having serious diseases of other systems; and having disorders that affected cognitive function. Healthy controls were included if they were between the ages of 18 and 65 and had no reported history of neurological or psychiatric disease. All participants were right-handed and had normal or corrected-to-normal vision. To avoid the influence of confounding factors that may also affect EC, the Montreal Cognitive Assessment (MoCA) for cognitive performance was administered.

### Behavioral paradigms

**Fig. 1:**
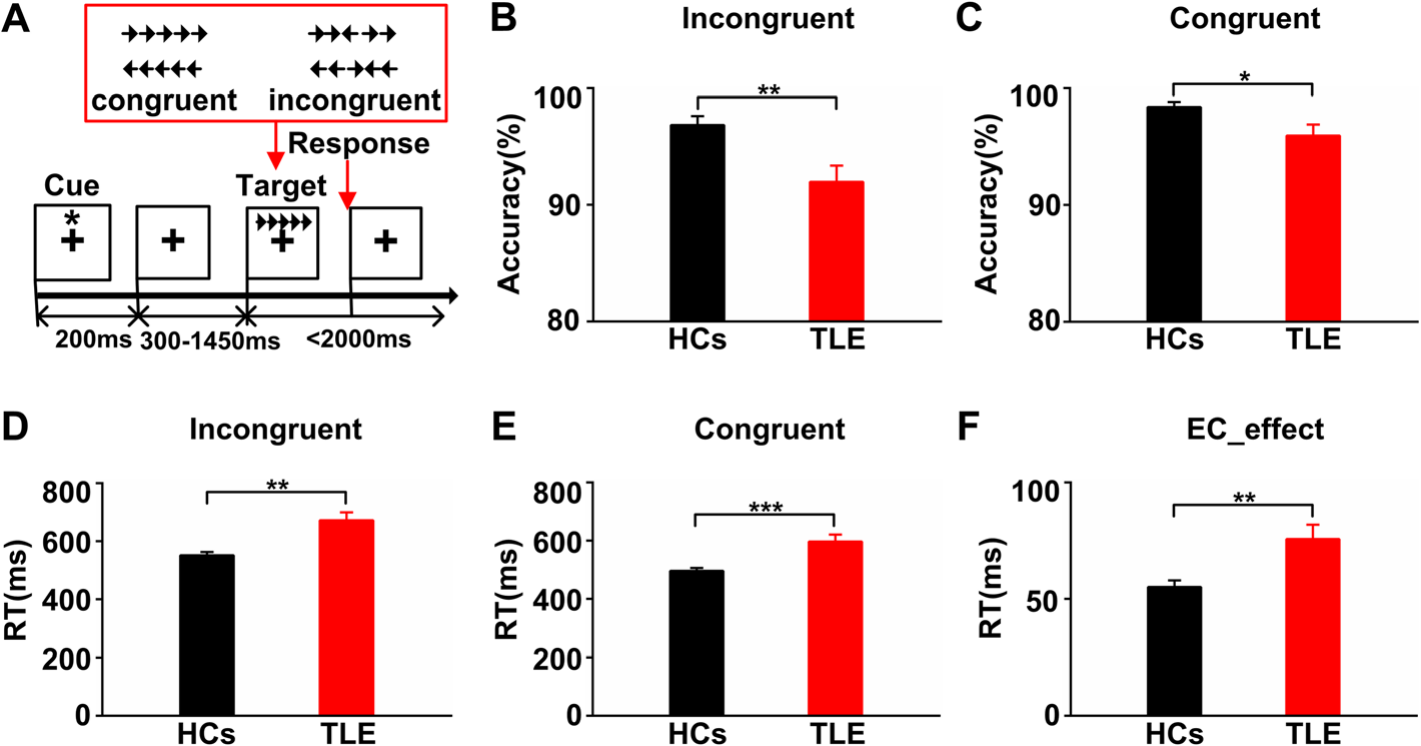
Behavioral performance. **a** Schematic of the test. **b** and **c** Averaged accuracy in the incongruent and congruent conditions. The accuracy was significantly lower for the TLE group compared to the HCs (t-test, *p* < 0.05). **d** and **e** Averaged RT in the incongruent and congruent conditions. The RT was significantly longer for the TLE group compared to the HCs (t-test, *p* < 0.01). **f** Performance for EC_effect in the EC tasks (EC_effect = RT_incongruent – RT_congruent). The EC_effect was significantly longer for the TLE group compared to the HCs (t-test, *p* < 0.01). Error bars indicate SEM

We adopted the attention network test (ANT) procedure designed by Fan et al. and established by E-prime 2.0 (Fig. 1a) [29]. There was a “+” in the middle of the screen and it was maintained throughout the whole experiment. The stimulus information consisted of a row of 5 directional arrows, including the target stimuli (the center arrow) and the flanks (two arrows on both sides of the target arrow). The directions of the four arrows on the flank were always consistent, and the direction of the target arrow was congruent (congruent task) or incongruent (incongruent task) with the flank arrows. At the beginning of each trial, one of three types of cues (no cue, center cue and spatial cue) appeared randomly and maintained for 200 ms. After a variable duration (300-1450 ms, mean = 550 ms), the stimulus was presented, and the subject needed to make a response based on the direction of the target. The stimulus information disappeared after the subjects responded, or appeared for 2000 ms. The average time of each trial was 4050 ms. Each subject completed a total of 6 blocks of trials (consisting of 6 buffer trials and 108 formal trials), with each block at least 5 min apart. In this study, EC is the ability to resolve conflicts, so the operational definitions of the EC effect based on reaction time (RT) were as follows:

1 $$ EC\_ effect= RT\_ incongruent- RT\_ congruent $$

where EC*_effect* represents to conflict resolution ability in the EC network, *RT_incongruent* represents the mean response time in incongruent tasks and *RT_congruent* represents the mean response time in congruent tasks.

Trials with RT of 200 ms − 1000 ms and correct trials were constructed for the EC network.

### EEGs acquisition and preprocessing

Nicolet EEG (YZB / USA 2783–2011) was used to record 19-channel EEG signals, and the electrode positions were placed strictly according to the international 10–20 system. EEG data were acquired by the NeuroScan QuikCap referenced to Cz and digitized at 1024 Hz. All channels had impedances below 5 kΩ. In each trial, the fitting periods for the EC network were 300 ms preresponse to 300 ms postresponse. The rest state (RS) signals were collected from the EEGs in the subjects with quiet and closed eyes conditions. Each subject collected 10 min of EEGs data and randomly intercepted 20 pieces of data. Each piece of data was 600 ms in length, which is consistent with the length of the task state. The recorded signals were analyzed with MATLAB (2012a); first signals were up-sampled to 4096 Hz and then were filtered to remove frequencies below 0.5 Hz and above 100 Hz. The acquired signals were re-referenced by the averaging values from all channels. Eye movements and myoelectricity artifacts were removed from the original recording EEGs by EEGLAB toolkit in MATLAB.

### DTF connectivity calculations

Granger causality analysis method can be used to analyze the direct interaction between multiple variables and it has been widely used in the field of computational neuroscience. The FC calculation estimators were performed through the multivariate autoregressive model (MVAR), and the DTF is a refined method based on MVAR, which was regarded as a useful method for causal connections analysis in the field of computational neuroscience [30]. In the framework of the MVAR model, the DTF was formulated. Define the DTF from channel *j* to channel *i*, which represents the causal effect from channel *j* to channel *i* at frequency *f*, and the calculation formula is as follows:

2 $$ {\gamma}_{ij}(f)=\frac{{\left|H(f)\right|}^2}{\sum_{m-1}^k{\left|{H}_{im}(f)\right|}^2} $$

where *γ*_*ij*_*(f)* represents the ratio of the inflow from channel *j* to channel *i* to all the other inflows to channel *i*, *H* is the transfer matrices and *k* is the number of the channels. The mean DTF value is a direct measurement of FC strengths among EEGs.

Although the γ_*ij*_ value was not zero, which indicates that there was a connection between the channel *j* and *i* connection, the connection could be “false connections”. Therefore, we used an alternative method to test the significance of *γ*_*ij*_. First, the EEG signals of each channel were randomly scrambled, recombined into new data, and then the reconstructed data *γ*_*ij*_ was calculated. This process was repeated 1000 times, and the empirical distribution of *γ*_*ij*_ could be obtained. The order of the 1–19 channels corresponds to the electrodes Fp1, Fp2, F7, F3, Fz, F4, F8, T7, C3, Cz, C4, T8, P7, P3, Pz, P4, P8, O1 and O2.

### Functional connectivity strengths of the whole brain

The whole brain FC strengths (DTF_global) was the arithmetic mean of all the elements of the 19 electrodes corresponding to the DTF matrices, which could be an index directly describing the FC strengths of the EC network and RS network. The DTF_global was defined as follows:

3 $$ DTF\_ global=\frac{\sum_{j\ne \in K}{\sum}_{j\ne j\in K}\gamma ij}{k\left(k-1\right)} $$

where *k* denotes the number of channels, and *K* denotes the collection of all channels.

### Spatial distribution of the FC

*DTF*_*i*_ represented the FC strengths of channel *i*, which was defined as the mean value of all the strengths from channel *i* to other channels and those from the other channels to channel *i*. According to the DTF matrices of connectivity network, the *DTF*_*i*_ was defined as follows:

4 $$ {DTF}_i=\frac{1}{2\left(k-1\right)}\ \sum \limits_{\ne i\epsilon V}\left({\gamma}_{ij}+{\gamma}_{ji}\right) $$

where *γ*_*ij*_ is the average values of *γ*_*ij*_*(f)*, *V* is the set of channels in connectivity network and the *k* is the number of channels. In brief, the FC strengths of channel *i* is an estimator to estimate the influence of each channel through the connectivity network.

### Functional connectivity strengths among brain regions

To determine the key brain regions during the EC network, as well as FC strengths among brain regions, we divided 19 electrodes into 4 brain regions: frontal (F: Fp1, Fp2, F3, Fz, and F4), parietal (P: C3, Cz, C4, P3, Pz, and P4), occipital (O: O1 and O2) and temporal (T: F7, F8, T7, T8, P7, and P8). Calculate the FC strengths from region *l* to region *k* according to the following formula:

5 $$ {DTF}_{kl}=\frac{1}{MN}\ \sum \limits_{i\epsilon K}\sum \limits_{i\epsilon L}{\gamma}_{i j} $$

where *γ*_*ij*_ expresses the average values of *γ*_*ij*_*(f)* in the given frequency band. *M* is the number of channels of group *k* and *K* is the set of channels of group *k*. *N* is the number of channels of group *l* and *L* is the set of channels of group *l*. The FC strengths within *K* region *DTF*_*kk*_ is defined as:

6 $$ {DTF}_{kk}=\frac{1}{M\left(M-1\right)}\ \sum \limits_{i\epsilon K j\ne}\sum \limits_{i\epsilon K}{\gamma}_{i j} $$

where *γ*_*ij*_ means the average values of *γ*_*ij*_*(f)* in the given frequency band. *M* is the number of channels of group *k* and *K* is the set of channels of group *k*.

In this study, the causal network efficiency of the EC network was defined as follows:

7 $$ DTF\_ effect= DTF\_ incongruent- DTF\_ congruent $$

where *DTF_effect* represents the mean DTF values of the effect in the EC network, *DTF_incongruent* represents the mean DTF values in incongruent tasks and *DTF_congruent* represents the mean DTF values in congruent tasks.

### Statistical analysis

In the present study, the statistical analysis was executed by utilizing SPSS version 20.0 (SPSS Inc., Chicago, IL). We recorded 19 channels EEGs data from 40 volunteers (20 HCs and 20 TLE patients) while they performed the EC task and the RS data. Trials with RT of 200 ms − 1000 ms and correct trials were constructed for the EC network in the present paper. Data in the text and figures are expressed as means ± SEM. Statistical differences were evaluated by using t-test and ANOVA. Specifically, behavioral accuracy and RT were analyzed using t-test. Comparisons of FC strengths between the two groups were done by using t-test. Comparisons of FC strengths between the EC network and the RS network were done by using paired t-test. Comparisons of the characteristic brain regions were analyzed using ANOVA. Pearson correlations between FC, accuracy and EC_effect were also calculated though partial correlation analysis with age, sex and education as covariates. *P*-values are marked statistically significant as follows cases: *P < 0.05, **P < 0.01 and ***P < 0.001.

## Results

### Demographic and clinical data

The demographic data of the two groups and the clinical information of the TLE patients are presented in Table 1. No significant differences were found between the two groups in terms of age (*t* = − 1.75, *p* = 0.071). The distribution of gender across groups was comparable (χ2 = 0.1, *p* = 0.75). The mean MoCA score of HCs was 28.15 ± 1.63 and the range was 26–30; the score of TLE was 28.05 ± 1.39 and the range was 26–30. No significant differences were found in MoCA scores between the two groups. In addition, eight patients (5 left and 3 right) were pathologically identified as having hippocampal sclerosis.

**Table 1:** Group demographics of the TLE patients and the HCs

	TLE (*n* = 20)	HCs (*n* = 20)	Test value	*P*-value
Age (Mean ± SD)	29.30 ± 10.32	27.55 ± 7.05	^a^-1.75	0.54
Gender (Male/Female)	10/10	11/9	^b^0.1	0.75
MoCA (Mean ± SD)	28.05 ± 1.39	28.15 ± 1.63	^a^0.21	0.84
Age of epilepsy onset	19.50 ± 9.36 (2–50)			
Duration (years)	10.10 ± 10.84 (1–42)			
Type of seizures^*^	20			
Focal automatisms seizure	6			
Focal atonic seizure	3			
Focal clonic seizure	1			
Focal tonic seizure	3			
Focal autonomic seizure	2			
Focal emotional seizure	2			
Focal sensory seizure	3			
Seizure Frequency (times/month)	0.70 ± 0.96 (0–4)			
AED Therapy (n, %):				
Monotherapy	9 (45%)			
Polytherapy	11 (55%)			

*MoCA* Montreal Cognitive Assessment, *AEDs* Antiepileptic Drugs, *TLE* temporal lobe epilepsy, *HCs* healthy controls, *SD* standard deviation. ^a^ t value; ^b^χ2 valuel; ^*^According to ILAE 2017 seizure classification [31]

### Behavioral performance

ANT accuracy and RT were analyzed via independent-samples T test. Figure 1b, c shows the accuracy results. The incongruent accuracy for the HC group was 96.86%, and for the TLE group was 91.98%. The congruent accuracy was 98.40% for the HC group, and 95.96% for the TLE group. The accuracy of the HC group was significantly higher than that of the TLE group (*P* < 0.05). Figure 1d, e shows the RT results. The incongruent RT was 551.45 ± 11.48 ms for the HC group, and 672.40 ± 27.54 ms for the TLE group. The congruent RT was 496.28 ± 9.72 ms for the HC group, and 596.69 ± 24.08 ms for the TLE group. Both in the incongruent and congruent conditions, the RTs of the TLE group were significantly longer than those in the HC group (*P* < 0.01). Figure 1f shows the EC_effect results. The EC_effect was 55.16 ± 2.78 ms for the HC group, and 75.71 ± 6.17 ms for the TLE group (*P* < 0.05). Compared with the HC group, the EC_effect of the TLE group was significantly longer (*P* < 0.05).

### Functional connectivity among 19 channels EEGs during the EC tasks

**Fig. 2:**
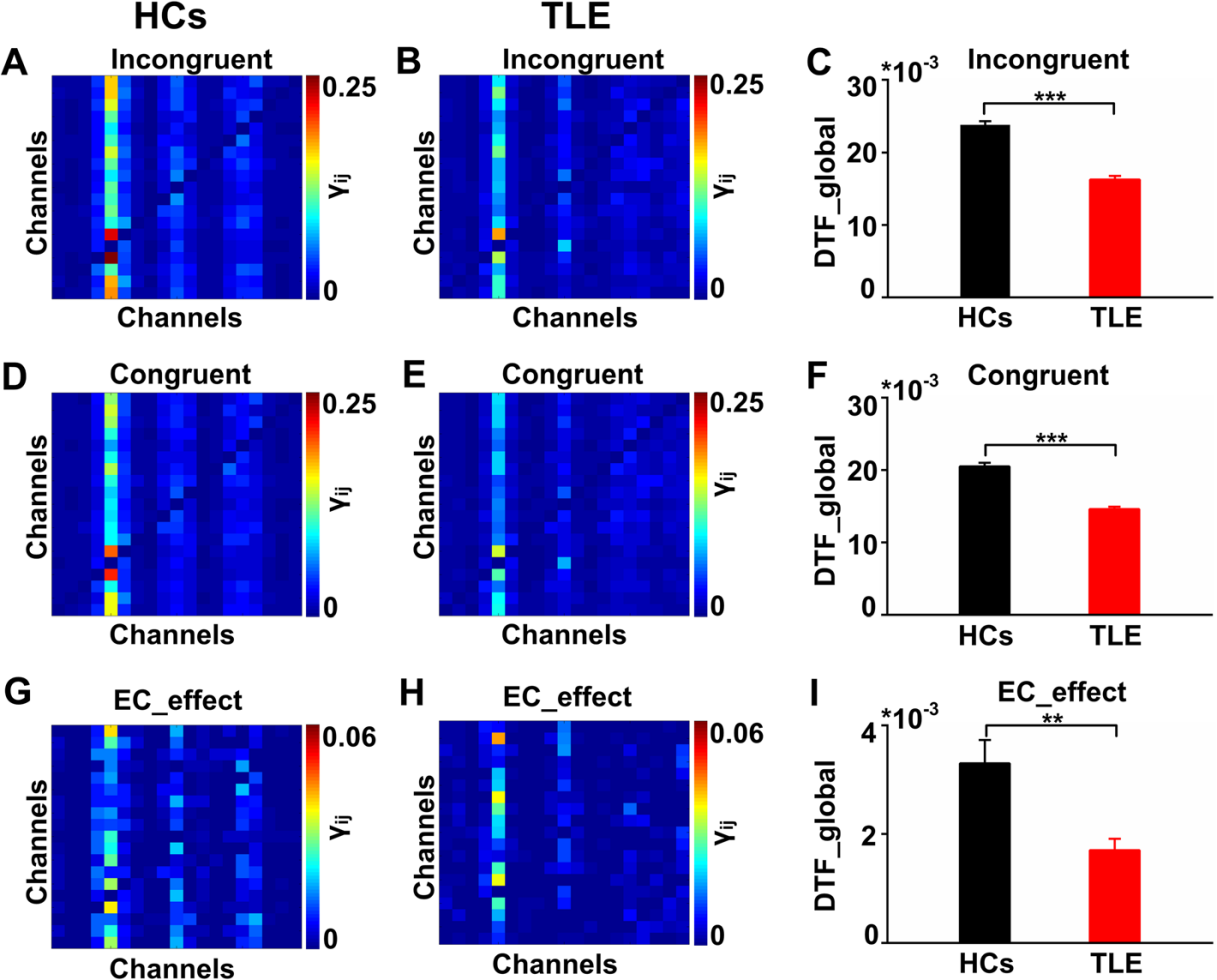
Functional connectivity among the 19 channels during the EC tasks. **a** and **b** Connectivity matrices of the 19 channels in the incongruent tasks in the two groups. The matrices show how much each node interacts one another. Each node represents a channel. The scaled colors represent the FC strengths from transverse channel to vertical channel. **c** Averaged DTF in the incongruent tasks in the two groups. Error bars reflect SEM. The DTF in the HCs was significant higher than that in the TLE group in the incongruent tasks (t-test, ***P < 0.001). **d** and **e** Connectivity matrices of the 19 channels in the congruent tasks in the two groups. **f** Averaged DTF in the congruent tasks in the two groups. The DTF in the HCs was significant higher than that in the TLE group in the congruent tasks (t-test, ***P < 0.001). **g** and **h** Connectivity matrices of the 19 channels of the EC network in the two groups. **i** Averaged DTF in the EC network in the two groups. The DTF in the HCs was significant higher than that in the TLE group in the EC network (t-test, **P < 0.01). The connectives were observed to primarily propagate from the frontal regions in both the HC and TLE groups. The FC strengths in the HCs were definitely higher than those in TLE group

We calculated the connectivity matrices in the theta band to estimate the FC strengths among EEGs during the EC tasks in the HCs and the TLE group. Exemplar connectivity matrices during the incongruent tasks are shown in Fig. 2a and b. In the incongruent tasks, the FC strength in the HC group (0.0238 ± 0.0005) was definitely higher than that in the TLE group (0.0163 ± 0.0005) (Fig. 2c, t-test, *P* < 0.001). Exemplar connectivity matrices during the congruent tasks are shown in Fig. 2d and e. In the congruent tasks, the FC strengths in the HC group (0.0205 ± 0.0005) was definitely higher than that in the TLE group (0.0146 ± 0.0003) (Fig. 2f, t-test, *P* < 0.001). Exemplar connectivity matrices in the EC network are shown in Fig. 2g and h. In the EC network, the FC strengths in the HC group (0.0033 ± 0.0004) was definitely higher than that in the TLE group (0.0017 ± 0.0002) (Fig. 2i, t-test, *P* < 0.01). The results showed that the DTF matrices of the two groups were mainly concentrated in the frontal region and parietal region. Moreover, in the EC network, the FC strengths in the TLE group were weakened than that in the HCs.

### Spatial distribution of the functional connectivity during the EC tasks

**Fig. 3:**
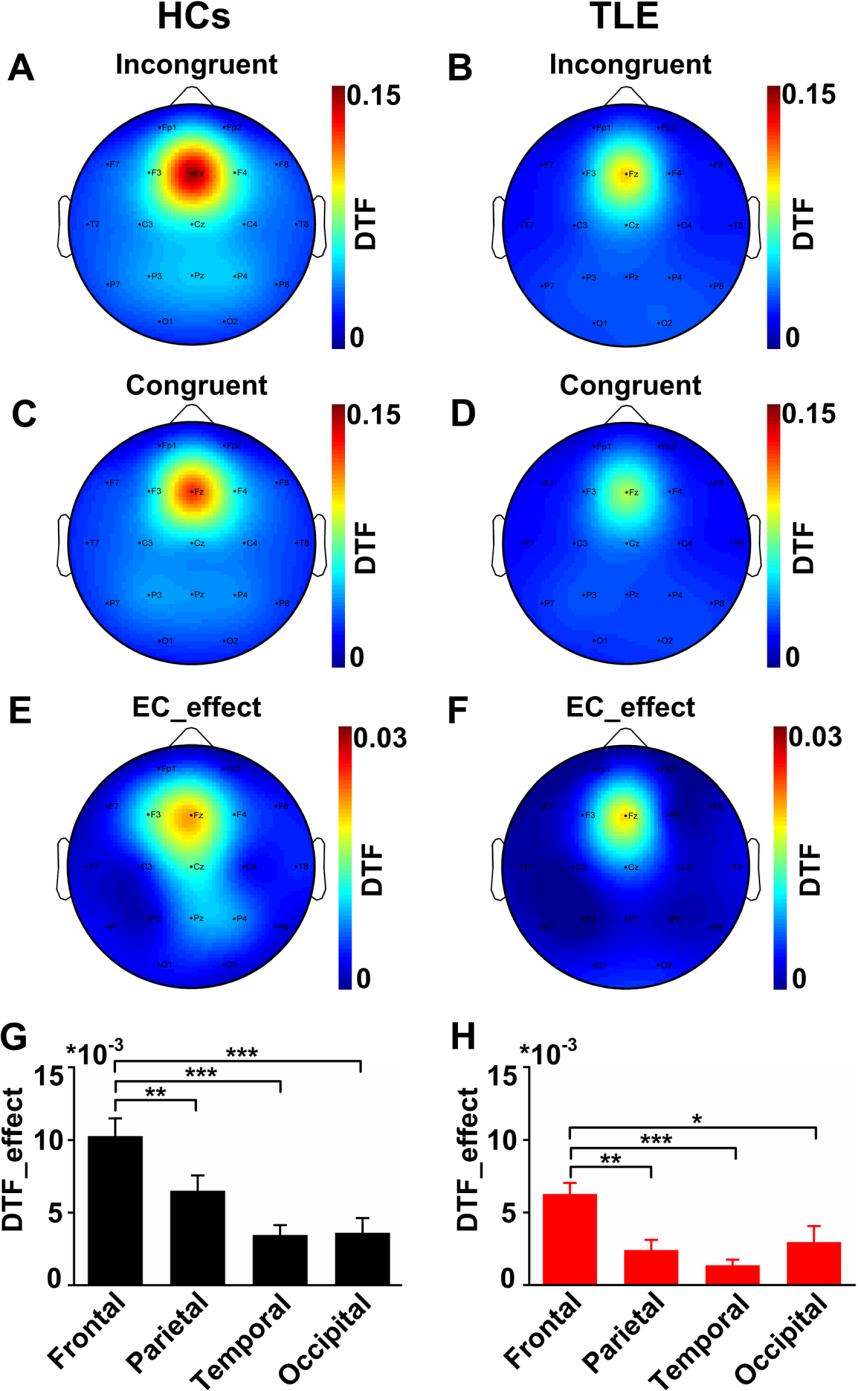
Spatial distribution of the functional connectivity during the EC tasks. **a** and **b** The topographical maps in the incongruent tasks in the two groups. The scaled colors represent the FC strengths. **c** and **d**) The topographical maps in the congruent tasks in the two groups. **e** and **f** The topographical maps of the EC network in the two groups. The spatial distribution of the FC in the three conditions was mainly concentrated in the frontal and parietal regions for both of the two groups. **g** and **h** Averaged DTF for the four brain regions in the two groups. Error bars reflect SEM The DTF of the frontal region was significantly higher than other brain regions both in the HC and TLE groups (ANOVA, *p* < 0.05)

To ascertain the strongest connectivity locations, a topographical map of the FC distributions in the EC network was calculated in the HCs and the TLE group. Figure 3a-f show the topographical maps of FC strengths for incongruent, congruent and EC_effect for the two groups. The DTF was strongest over the frontal midline region (Fz electrode) for each condition in both the two groups. Figure 3g and h show the averaged DTF histograms among the four brain regions in the EC network in the two groups, respectively. The DTF of the two groups of the frontal region was the highest among the four brain regions (ANOVA, *P* < 0.05). The results indicated that the frontal region was the characteristic brain region of the EC network.

**Fig. 4:**
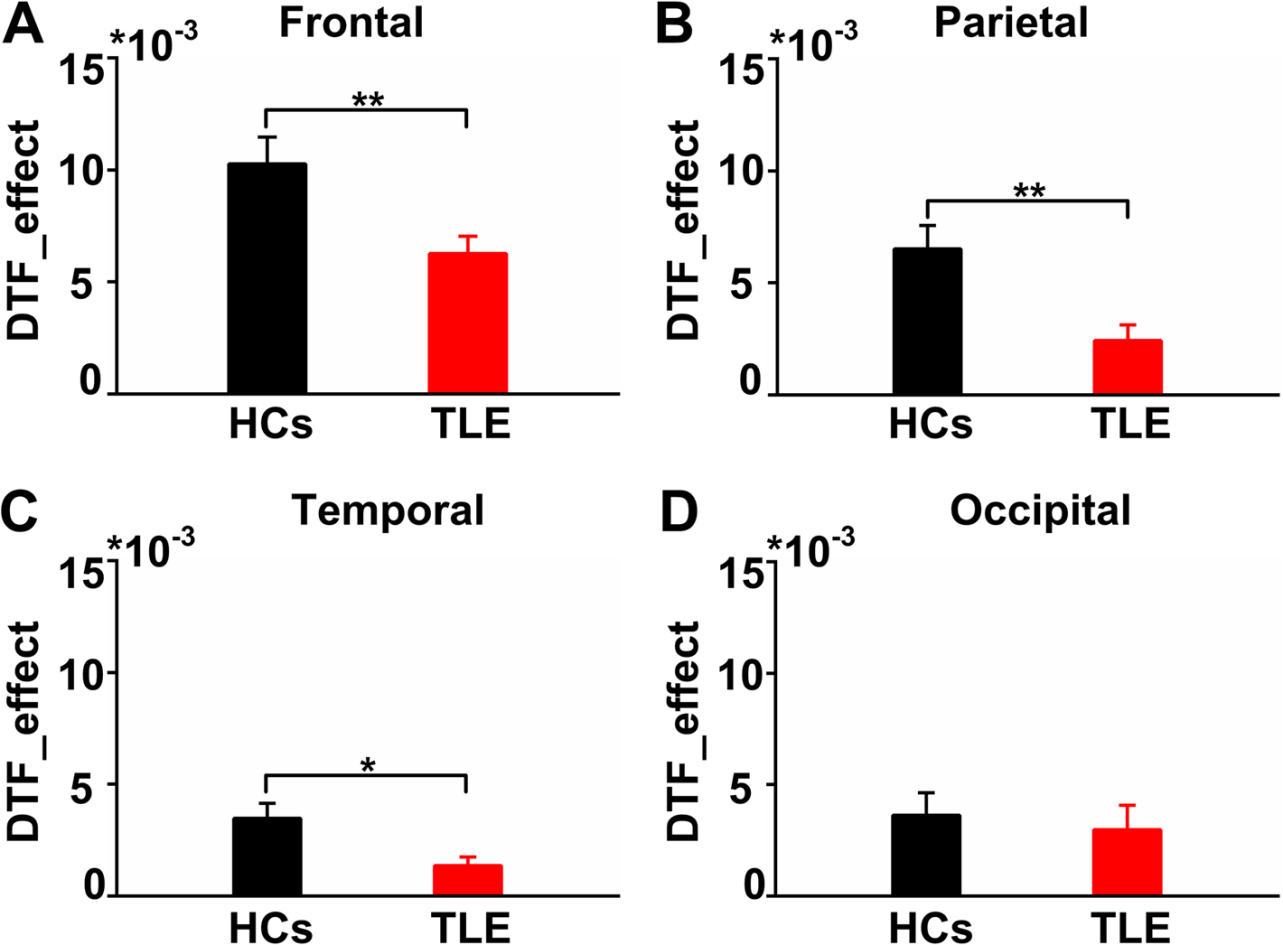
Functional connectivity of the fou brain regions during the EC tasks. Comparison of averaged DTF in **a** frontal region, **b** parietal region, **c** temporal region, **d** occipital region of the theta band. The averaged DTF in frontal region, parietal region and temporal region were significantly lower in TLE group than that in the HCs (t-test, *P < 0.05, **P < 0.01). Error bars indicate SEM

We then compared the FC strengths in the four brain regions between the two groups. As shown in Fig. 4, the FC strengths of the EC network in the TLE group were weakened in the frontal region (HCs: 0.0103 ± 0.0012, TLE: 0.0063 ± 0.0008) (t-test, *p* < 0.01), parietal region (HCs: 0.0065 ± 0.001, TLE: 0.0024 ± 0.0007) (t-test, *p* < 0.01) and temporal region (HCs: 0.0035 ± 0.0007, TLE: 0.0014 ± 0.0004) (t-test, *p* < 0.01) (t-test, *p* < 0.05).

### Functional connectivity strengths within and among frontal region

**Fig. 5:**
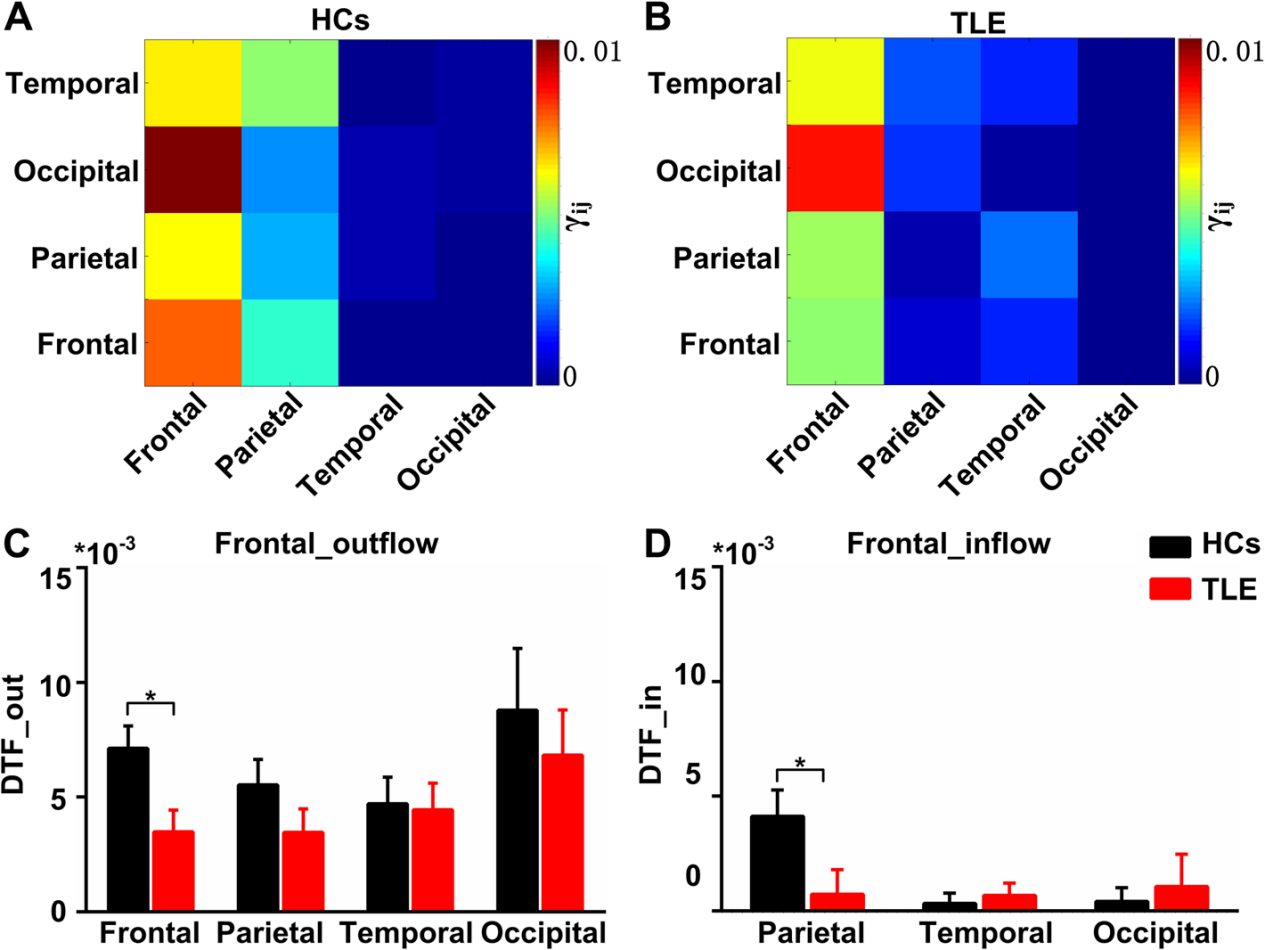
Functional connectivity strengths among the 4 brain regions during the EC tasks. **a** Connectivity matrices among the four brain regions in the EC network in the HCs. The matrices show how much each brain region interacts one another. Each node represents a brain region. The scaled colors represent the FC strengths from transverse brain region to vertical brain region. **b** Connectivity matrices among the 4 brain regions in the EC_effects in the TLE group. The connectives in the frontal region clearly increased both in HCs and TLE patients. The DTF of the frontal region in the HCs were definitely higher than those in TLE group. **c** Comparison of the FC strengths from the frontal region outflow between the two groups. The DTF from frontal region to frontal region in the HCs was significant higher than that in the TLE group (t-test, *p* < 0.05). **d** Comparison of the FC strengths from the frontal region inflow between the two groups. The DTF from parietal region to frontal region in the HCs was significant higher than that in the TLE group (t-test, *p* < 0.05). Error bars indicate SEM

Considering the FC strengths was strongest over the frontal midline region, we calculated the FC strengths from frontal to frontal, frontal to parietal, frontal to occipital, frontal to temporal, parietal to frontal, occipital to frontal and temporal to frontal. Figure 5a and b show the averaged DTF matrices of the EC_effects for the four brain regions in the HC and TLE groups. Figure 5c shows the DTF from the frontal region to other brain regions (frontal_outflow) and Fig. 5d shows the DTF from other brain regions to frontal region (frontal_inflow). As can be seen, the TLE group exhibited significantly decreased FC in frontal_outflow (from frontal region to frontal region) and in frontal_inflow (parietal region to frontal region) compared with the HC group during the EC tasks (t-test, *P* < 0.05).

### Functional connectivity strengths in the RS network

**Fig. 6:**
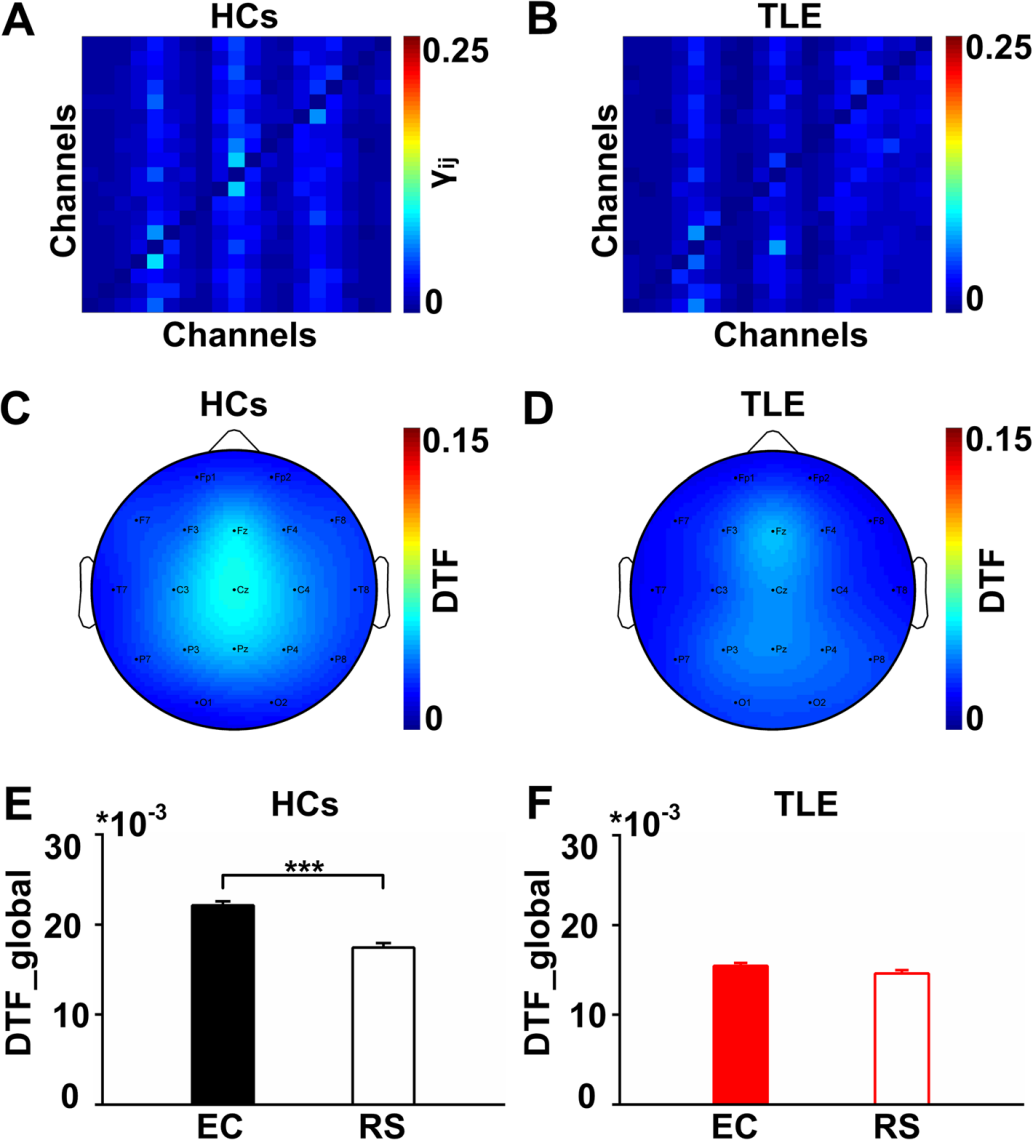
Functional connectivity in the EC network and RS network. **a** and **b** Connectivity matrices of the 19 channels in the RS network in the two groups. **c** and **d** The topographical maps in the RS network in the two groups. The FC strengths in the RS network were definitely weaker than those in the EC network. **e** Comparison of averaged DTF between the EC network and RS network in the HCs. The averaged DTF in the EC network was significantly higher than that in the RS network (paired t-test, *P* < 0.001). **f** Comparison of averaged DTF between the EC network and RS network in the TLE group. The averaged DTF in the EC network and RS network showed no significant difference (paired t-test, *P* > 0.05)

In order to further verify that the FC strengthened is directly related to the EC network, this study compared the FC strengths of the EC network and RS network both in the HC and TLE groups. Exemplar connectivity matrices during the RS in the HCs and TLE group are shown in Fig. 6a and b. Figure 6c and d show the topographical maps of FC strengths for the RS network in the two groups. In the HCs, the FC strengths in EC network (0.02217 ± 0.0004) is significantly higher than that in RS network (0.0175 ± 0.0005) (paired t-test, *P* < 0.001). However, there was no significant change in FC strengths between the EC network (0.0155 ± 0.0004) and the RS network (0.0146 ± 0.0004) in the TLE group (paired t-test, *P* > 0.05). Since the increasing trend of FC strengths were found in the HCs during EC tasks, it is considered that the enhanced FC is indispensable for information processing in EC function. The FC strengths of the EC network in TLE group is not enhanced compared to the RS network, which may be the cause for the EC deficit in the TLE patients.

### Correlation analysis of FC with behavioral performance

**Fig. 7:**
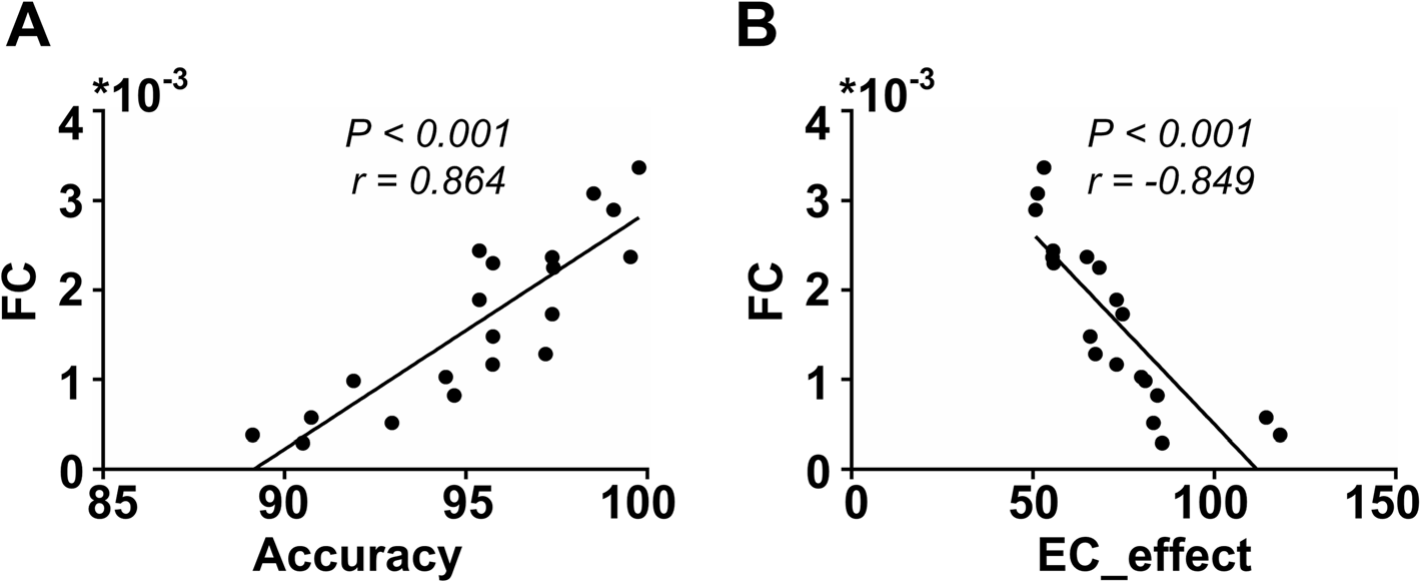
Correlation of FC with behavioral performance: FC had positive correlation with accuracy (*p* < 0.001, *r* = 0.864) and negative correlation with EC_effect (*p* < 0.001, *r* = − 0.849)

To further validate the association between FC decreased and behavioral performance in TLE patients, Pearson correlations between FC and behavioral indicators were calculated in this study. As shown in Fig. 7, we found that the FC was significantly positively correlated with accuracy (*p < 0.001, r = 0.864*) and negatively correlated with EC_effect in TLE group (*p < 0.001, r = − 0.849*).

## Discussion

Our results show that the FC strengths in the HCs increases during the EC task. In contrast, the FC strength in the TLE group was relatively weaker and did not significantly change when compared to RS network. Moreover, we demonstrated that the frontal region was the principal brain region in the EC network. Significantly decreased FC strengths were found in the theta band of the frontal region in TLE patients compared to the HCs. In addition, in the TLE group, we found that the functional connectivity was significantly positively correlated with accuracy and negatively correlated with EC_effect. The results propose that the strengthened FC is necessary for information manipulation in EC and the decreased FC strengths in TLE group may cause the EC impairment in TLE patients.

The EC is considered as a high level of cognitive processing which is mainly dependent on the frontal lobe function. In this study, we calculated the FC strengths for each brain region and showed that the strongest FC region was found in the frontal midline region, confirming the importance of the frontal lobe in EC, which is consistent with other neuroimaging findings on EC [32–35]. Our results also indicate that the FC strength in frontal region of TLE group was weaker than that of HCs, which may be the reason why TLE patients show deficiencies in RT and accuracy during EC tasks. This suggests that the EC impairment in TLE follows a pattern of neural dysfunction in the cognitive network, which is not typically associated with temporal lobe dysfunction, but may be associated with frontal dysfunction [36–38]. For example, Keller et al. used quantitative MRI to study the EC of TLE, and the results showed that unilateral TLE had volume atrophy of ipsilateral hippocampus and bilateral prefrontal cortex [39]. Takaya et al.’s study showed that after selective amygdalohippocampectomy, glucose metabolism increased in extratemporal areas ipsilateral to the affected side, such as the dorsolateral prefrontal cortex, dorsal medial and ventral medial cortex. In addition, postoperative verbal memory, delayed recall and attention/concentration scores were significantly better than preoperative scores, which suggested that cognitive function in TLE patients was associated with glucose metabolism in the frontal lobe [40]. Justus et al. used MRI and [^18^F] fluorodeoxyglucose positron emission tomography (FDG-PET) to study the memory of the TLE, and the results showed an interactive negative effect of metabolic and structural temporal lobe abnormalities on verbal memory [41]. These findings were consistent with the hypothesis of previous studies that structural and metabolic abnormalities were involved in TLE patients with cognitive impairments [42, 43]. Our findings add to the mounting evidence of neuroelectrophysiology for extratemporal cognitive deficits in TLE.

In addition, our results also showed that the FC in the temporal outflow of TLE group was more than that of HCs (Fig. 5b). The possible reason is that the temporal lobe is the focus of abnormal discharge of epilepsy, and there are many pathological wiring, but it does not compensate for cognitive curtailing. The results of Dinkelacker and her colleagues confirm our hypothesis that increased connections in the temporal lobe is associated with decreased EC function in TLE patients [44].

Neural oscillations play an important role in various cognitive functions [45]. Different physiological states determine different oscillation modes, and different cognitive processes (EC, attention, learning, and memory) have distinct neural oscillation modes [46, 47]. In this study, we used the DTF of the inconsistent condition minus the DTF of the consistent condition to eliminate the influence of irrelevant information in the EEGs. Fan et al. also introduced a subtraction mode in the neural oscillations of the attention network, and found that different frequency bands participate in different attention processes [29]. This subtractive mode eliminates the effect of resting state on neural electrical activity in cognitive processes. Therefore, the experimental results are more sensitive to the FC changes caused by cognitive activity itself.

Theta activities are broadly distributed across the brain and appear to reflect active operations of the generative cortex, particularly during high-level cognitive processes such as memory encoding, novelty detection, and realizing the need for top-down control [48, 49]. It was well established in the previous studies that the frontal theta activity is increased during EC test [28, 47]. Kristin et al. found that the long-range FC between the prefrontal cortex and posterior parietal cortex was mediated by neuronal oscillations of the theta band during sustained attention [47]. Julia et al. found that the decline in fronto-parietal FC of theta band was a neurological mechanism in patients with goal-directed attention disorders [50]. In this study, we found that the strengthened theta connectivity was closely related to the EC network while performing the ANT. This finding is consistent with the belief that the theta synchronization activities in prefrontal cortices emerged during goal_directed behavior tests [51–54]. Thus, decreased FC in theta band may contribute to the EC deficits in TLE patients.

### Limitations

A number of limitations to our study need to be considered. Firstly, the sample size of each group in our study is small, and it is possible to conduct more extensive analysis with the increase of sample size. For example, we can subdivide the TLE into the left TLE and right TLE groups for statistical comparison. In spite of our limited sample size, we were able to document these novel findings. The results of this study show that the characteristic brain region of the EC network is the frontal region, while the results of other scholars have also shown that left TLE and right TLE patients have frontal cognition dysfunction [36, 39, 43]. This study aimed to explore the neural mechanisms of frontal lobe dysfunction in TLE patients. Therefore, in the experimental design, this study did not subdivide left TLE and right TLE.

Secondly, the DTF is included in effective connectivity, which is directional. One of the shortcomings of this study is that the information flow between the channels is not further calculated in this study. However, our results show that the frontal region is the characteristic brain region of the EC network. In this study, DTF values in and out of the frontal lobe were calculated to determine the pattern of impaired executive control network in TLE patients. Future studies using larger sample sizes are necessary to characterize the brain network defect mechanism of EC dysfunction in TLE patients, which in turn will further understand the mechanism of typical “frontal lobe” dysfunction in TLE patients.

## Conclusions

In conclusion, our results demonstrated that the FC strengths of the theta band in HCs increased during EC network while the FC strength in the TLE patients was relatively weaker. It is assumed that the increased FC strength in the theta band is necessary for information manipulation in EC; thus, the decreased FC strengths may provide a potential mechanism for EC deficits in TLE patients. From the perspective of functional brain network, the neural mechanism of TLE comorbidity to EC deficits is clarified in depth, which provides a new theoretical basis and innovative research ideas for understanding the pathogenesis of TLE comorbid cognitive dysfunction.

## Data Availability

Laboratory policy restricts availability of the raw data and materials to the investigators due to the inherent complexity and volume of raw EEG data and the unique, complex file structures for such data storage and associated data analyses.

## Abbreviations

TLE: Temporal lobe epilepsy
FC: Functional connectivity
EC: Executive control
ANT: Attention network test
EEG: Electroencephalogram
HCs: Healthy controls
DTF: Directed transform function
RT: Response time
SEM: Standard error of the mean
MRI: Magnetic resonance imaging
